# Knowledge gaps and national research priorities for COVID-19 in Iran

**DOI:** 10.1186/s12961-021-00805-y

**Published:** 2022-03-02

**Authors:** Bahareh Yazdizadeh, Elham Ehsani-Chimeh, Kazem Zendehdel, Mohammadreza Mobinizadeh, Bita Mesgarpour, Zeinab Fakoorfard

**Affiliations:** 1 Epidemiology, Knowledge Utilization Research Center, Tehran University of Medical Sciences, Tehran, Iran; 2 Health Services Management, National Institute for Health Research, Tehran University of Medical Science, Tehran, Iran; 3 Epidemiology, Cancer Research Center, Cancer Research Institute, Tehran University of Medical Sciences, Tehran, Iran; 4 Pharmacoepidemiology, National Institute for Medical Research Development (NIMAD), Tehran, Iran; 5 Health Economics, National Institute for Health Research, Tehran University of Medical Science, Tehran, Iran

**Keywords:** COVID-19, Knowledge need, Research priority-setting, Challenges, Research priority, Health systems research

## Abstract

**Background:**

In the present COVID-19 crisis, one of the greatest challenges for research funding at both the international and national level is selecting the best research topic to achieve efficiency and equity in health research and to address the knowledge gap urgently raised due to the event. Despite international recommendations, countries should consider their context-specific situation and define local research priorities. We aimed to exercise a priority-setting activity to identify the knowledge gaps and suggest research priorities in response to the COVID-19 epidemic in Iran.

**Methods:**

First, we tried to identify the contextual knowledge gaps based on an online survey, performing key informant interviews (i.e. health professionals, policy-makers and managers) and media analysis. We also performed a literature review and considered international research priorities for COVID-19. Subsequently, we prepared a list of research questions and challenges to respond to the COVID-19 crisis in Iran using a systems approach. Then we mapped approved COVID-19 research projects in the country to research questions. Finally, we compared the identified research questions (not challenges) with the prioritized research from international organizations and then prioritized them for Iran.

**Results:**

We found risk factors and epidemiological dissemination patterns of the virus and its consequences in an *epidemiology domain*, implementation of clinical and hygiene in a *clinical management domain*, genetic studies for targeting prevention and treatment in a *candidate treatment and vaccine research and development (R&D) knowledge domain*, examination of the manifestations of ethics in society instead of ethics in research in an *ethics domain*, “care, access and health system” and “public health and participation in response to public health and clinical research” as two sub-domains of a *social sciences domain*, and finally, no new questions in either the *virology, transmission, diagnosis* or *animal and environmental domain*.

**Conclusions:**

In the event of global health crises like COVID-19, prioritization of research questions can be done globally, but some of the research priorities are context-specific and may vary by regional needs. To better manage research resources, researchers must respond to the challenges faced in each country based on its political, economic, social and cultural characteristics, and to make evidence-informed decisions, global knowledge gaps must be customized in each country.

**Supplementary Information:**

The online version contains supplementary material available at 10.1186/s12961-021-00805-y.

## Background

Health research funding organizations have always faced the question of what research to fund to maximize efficiency for themselves and their community. Therefore, in evaluating a research system, efficiency (compared with the costs, are the impacts of the research acceptable) and equity (is the research based on the needs of society) are typically considered in addition to effectiveness (whether the research has led to the desired impact) [1]. The COVID-19 virus epidemic has cornered research funding organizations in a new tight spot by creating an urgent need to answer new questions, along with a persistent lack of funding for research in some countries.

In the context of the COVID-19 epidemic, there is an urgent need for knowledge to understand the epidemiology of the disease, disease transmission and prevention strategies, vaccines, clinical characteristics of the patients, appropriate treatments and new technologies, socioeconomic factors of the patients, behavioural and psychosocial issues, and governance of the health system and making effective policies for the prevention and control of the disease at the regional, national and international levels [2].

Research system efficiency is related to reducing the waste of research resources, and equity is related to research prioritization. Research priority-setting exercises will help increase the efficiency of the research system [3] and improve the resilience of the health system in responding to the COVID-19 epidemic and similar crises in the future [4]. For these reasons, COVID-19 research priority-setting was considered at the international and national levels.

World experts on COVID-19 met at the WHO headquarters in Geneva in collaboration with the Global Research Collaboration for Infectious Disease Preparedness (GloPID-R) on 11–12 February 2020 to collect and assess the current knowledge about COVID-19 and reach a consensus on critical research questions that needed to be answered. This road map aimed at a proper diagnosis and optimum care for affected people, prevention of COVID-19, and supporting research priorities that would lead to the development of a global research platform. Eight immediate research actions, a crosscutting, interdisciplinary approach, and practical implications with international solidarity were suggested in this road map [2, 5].

To apply these research priorities, each country should keep the following considerations in mind:

- There are some knowledge needs that are relevant to all countries, and their response to them can be generalized and applicable for all countries.
- There are some knowledge needs for which the answers are different for different countries, and the countries themselves must find the answers to those needs.
- There are some knowledge needs that are specific to each country and have not been identified by the international organizations’ research prioritization.

Iran has 64 medical universities/schools with 20 966 academic staff and 755 health and biomedical research centres [6], with a growing need for and interest in COVID-19-related research worldwide. Iranian researchers and the scientific community became motivated to enhance their impact in combatting the COVID-19 epidemic. The Vice-Chancellor for Research and Technology at the Ministry of Health and Medical Education and the National Institute for Medical Research Development (NIMAD) in Iran decided to support research projects that would answer the national knowledge gaps and answer the research questions that are both novel and applied in a national and international context during the first 3 months of the epidemic. We then designed this study to identify the knowledge needs for which responses were required to address the COVID-19 epidemic in Iran, prevent such crises in the future, increase health system resilience in the face of future epidemics, and find the necessary considerations when we use international organizations’ research priority-setting.

## Methods

This study was conducted during the first 3 month of the COVID-19 epidemic in Iran (first wave) from 20 February 2020 until 20 May 2020 to identify the mentioned knowledge needs. We followed a two-step procedure to determine the contextual knowledge needs and define research priorities.

### Stage 1: Extracting knowledge needs at the national level

Three different methods were used to identify the knowledge needs for controlling COVID-19; the details of each method are described below. The main approach to finding the context-specific knowledge gap was identifying challenges in the response to COVID-19 in the country. We believed that for addressing these challenges, we must investigate each barrier to respond to four groups of questions: (1) the magnitude of the challenge, (2) the root causes of the challenge in the system, (3) the mechanism by which the challenges affect the response to COVID-19 and (4) the solutions for resolving the challenge.

#### Method 1: Online survey

An online questionnaire was designed, and research subjects were collected from professional groups and researchers. This questionnaire link was posted on the websites of both the Research and Technology Department at the Deputy of Ministry of Health and Medical Education and the National Institute for Medical Research Development (NIMAD), its address link was announced through professional social media groups and news reporters, among others, and people were encouraged to complete it. In this questionnaire, the respondents were first asked, “What do you think about the most important challenges of controlling the current epidemic? Also, what research questions should be answered to control and manage the COVID-19 epidemic in the country and prevent similar events?” They were then asked to state their top five priorities. A total of 162 persons completed this survey, of which about 80% were directly involved in COVID-19 because of their job.

#### Method 2: Interview with the stakeholders

Semi-structured interviews were selectively conducted with policy-makers and senior managers, healthcare providers, health executive managers, faculty members and health researchers. Due to time constraints and problems in accessing individuals, we used convenient sampling to select the interviewees. We tried to recruit individuals from different provinces. The interview guide included two main questions: What were the challenges of managing and controlling the country’s epidemic, and which research is needed to manage and control the country’s epidemic?

#### Method 3: Media analysis

Analysis of news and social media groups was performed to identify challenges from the public perspective. The news agencies were purposefully selected from the two groups of government news agencies and independent news agencies. One government news agency and five independent news outlets were also chosen from the pages of individual and social virtual networks of activists in this field. The review time was between 4 February and 20 March 2020.

Media news was searched with the keywords COVID, corona, Wuhan China disease, and coronavirus, and related news was extracted. The news was then selected for further review that met the following criteria:

1. The positive and negative effects of COVID-19 in Iran and worldwide
2. Concerns, objections, opinions and challenges of people about COVID-19
3. Questions and research ideas about COVID-19.

For each news item, the date, headline, summary, name of the organization/reporter, link and source of the data were extracted and recorded.

##### Data analysis method

To analyse the data obtained from these three methods, a manifest content analysis approach was used [7]. In media analysis, after extracting the news, all news summaries were analysed and coded by two independent researchers. One of them is a specialist in social media (MH), and the other one in health policy and management (EE). To avoid missing any data, all summarized data were included in the study. In these data (gathered from the abovementioned methods), two main categories were identified from the beginning; the first one was needed research questions, and the other was existing challenges, and each went through a separate path. All challenges which were expressed were related to the health policy and systems research (HPSR) field. Therefore, according to the two variables (questions or challenges) and research area, we developed a matrix in which we put research areas A and B in rows and research questions and challenges as variables in columns (Table 1). In this matrix, the cell representing the intersection of research area A and challenges remained blank. The research areas were defined as outlined below:

**Table 1:** Categorization of needed research questions and challenges

Research area	Challenges	Research question
Research area A: Research areas introduced by GloPID-R include virology, disease transmission and diagnosis, the role of animals and the environment, epidemiology, clinical management, infection control, evaluation of the effectiveness of treatments, and vaccine production	No challenge was expressed by the participants in these areas	✓
Research area B: HPSR and areas of ethics and social sciences from the GloPID-R classification	✓	✓

Research area A: Research questions that were asked directly by individuals were added to the list of GloPID-R priorities [8]. This list was then quantitatively prioritized (stage 2).

Research area B: In this area, research questions and challenges were expressed independently by the participants. Independent means that participants articulated challenges and research questions without relating them, some referring to both and some referring to one. Research questions were added to the list of GloPID-R priorities. The challenges expressed by the participants were analysed and classified according to National Public Health Performance Standards [9]. Finally, HPSR and areas of ethics and social sciences from the GloPID-R classification in this area were addressed.

### Stage 2: Prioritizing the knowledge topics

We used two steps to select and prioritize knowledge needs.

#### Step 1: Comparing the results of the first part with the ongoing projects in the country

At the time of this study, medical universities in Iran had started researching COVID-19. The titles of the approved projects in Iran during the period from 1 March 2020 to 20 April 20 2020 were extracted from the National Research Ethics Committee website (http://nimad.ac.ir/content/200/COVID-%DB%B1%DB%B9-Priorities) and were then adapted to the proposed priorities by GloPID. One of the team's researchers who is familiar with medical science (BY) made this adaptation. This adaptation was then reviewed and modified by two specialists in clinical and basic clinical sciences. The purpose of this step was to prevent rework and identify needs for which appropriate research is not being conducted.

Then, the research team and representatives of NIMAD (as the most important stakeholder of the prioritizing list) eliminated several research questions. The exclusion criteria at this stage were two main variables: the mission of NIMAD (the research topic about vaccine was then excluded because it was funded by another funding agency) and the adequacy of ongoing research projects, mainly which caused the elimination of the research questions related to the identification of main and alternative therapies and the infeasibility of conducting those research projects in Iran. Finally, the prepared list was used in the quantitative section (step 2).

#### Step 2: Prioritizing research questions quantitatively

The list of research questions in areas A and B were included in this step. Then, in order to value the research questions, we used three indicators for the decision, including importance, feasibility according to the human resources and feasibility according to the physical resources.

The checklist of research questions was provided to the health system’s executive and academic experts in the form of a questionnaire. These experts comprised the National Research Committee for COVID-19 and a few Ministry of Health Deputy of Research managers; 17 experts participated in this stage. The experts were asked to assign a score between 1 and 3 to the specified titles according to the definitions provided for each of the indicators, and by performing a simple average, their opinions were summarized in a matrix.

Finally, based on the experts’ evaluation and the weights obtained from Shannon entropy method, the following research priorities were extracted through a multi-criteria decision-making model (MCDM). The methodological details of this part are presented in Additional file 1: Appendix S1.

## Results

The results are presented according to the research questions and their prioritization (group A) and then the identified challenges for responding to the COVID-19 epidemic (group B).

The identified research questions are presented in Table 2 (starred questions). No new questions were identified in virology, transmission or diagnosis. No new questions were identified in the domain of animal and environmental research on the origin of the virus and management measures regarding the link between humans and animals.

**Table 2:** Research priorities based on final ranking

Domain	Title/subject	Final ranking
Epidemiology	*The comparison and evaluation of the differences in the hospitals over time in terms of symptom severity, hospitalization rate, length of intensive care unit (ICU) hospitalization, type of treatment and quality of services provided, death rate in ICU, death rate outside ICU, duration of hospitalization in ICU and comparison of mortality rates after adjusting for age, sex, comorbidity and other confounding variables	1
Epidemiology	*Calculating the economic burden of the COVID-19 disease in the country	2
Social science, public health and participation in responding to public health and clinical research	*Assessing the acceptance of interventions by people (why do people not stay at home?). Analysing people’s preferences in accepting home quarantine, especially in the final period of the outbreak where the number of new cases is low but the risk of identifying positive cases and contagion is essential for the next wave	3
Epidemiology	*Studies on prevention and studies on people's behaviour (in the workplace, shops, parks) in relation to physical distancing methods and in consideration of health recommendations	4
Social science, public health and participation in responding to public health and clinical research	*Identifying methods to reduce the psychological burden (stigma) resulting from a positive COVID test	5
Epidemiology	*Comparison of provinces, cities and even urban and rural areas in terms of preparedness, speed, social and private sector participation, hospitals’ preparedness, and disease prevention and management and its relationship with disease outbreak and mortality	6
Clinical management	*Evaluate the problems and management methods (diagnosis, treatment and follow-up) of non-COVID patients who are in critical condition	7
Epidemiology	*Analysing the factors affecting the epidemiological trend of the disease in Iran/investigating the causes of epidemic differences and clusters created at different levels, including provinces as well as cities, villages, domains and areas within different provinces and cities, nursing homes and work environments, and analysing its causes within the framework of epidemiological studies/detailed study of similarities and differences of disease clusters in specific populations such as nursing homes and work environments	8
Epidemiology	*Recording the documents and data for COVID-19 patients with the aim of evaluating diagnostic methods, disease progression, interventions and drugs used, intensive care and consequences for COVID-19 patients at the national and provincial levels	9
Epidemiology	Outbreak assessment with different methods (direct, indirect, laboratory methods and questionnaire) in different parts of the country	10
Epidemiology	*Geographical distribution of patients who attended hospitals and investigation of the problems in the management of the distribution of patients in city hospitals by university headquarters	11
Clinical management	*Evaluate and compare hospital management challenges and initiatives, including the extent of hospital preparedness, management of medical and nonmedical personnel, healthy nutrition, infection control protocol, volunteers, equipment supply, transportation of corpses, non-COVID hospitalized patients, staff stress, personnel protection, education, incentive system, etc. and their impact on hospital performance	12
Epidemiology	What social distancing interventions have been most effective in preventing or reducing the spread of COVID-19? If children are less susceptible to the disease or not transmitting the disease, should schools remain closed? *Is physical distance necessary in this group? *How much did the closure of schools reduce contacts?	13
Social science, public health and participation in responding to public health and clinical research	What are the relevant, practical and effective approaches to increasing acceptance, absorption and adherence to public health interventions for the prevention and control of COVID-19, and how can side effects be identified and mitigated rapidly?	14
Care, access and health system	*Examining the psychological condition of the families of medical staff (or other occupations that have heavy responsibilities in the current situation, such as the police)	15
Clinical management	*Assessing the extent and severity of medical errors during COVID-19 crises and emergencies	16
Epidemiology	Which restraining societal interventions can better reduce the local spread of the disease? *Should quarantine be done or not? *How contacts changed during the New Year’s holidays?	17
Clinical management	*Checking the correctness and implementation of the presented protocols/checking the clinical aspects of the disease, including guidelines for managing patients with different degrees and severity of the disease	18
Epidemiology	*The relationship between occupational factors and COVID-19 incidence, especially occupations in the health sector, personnel, contractors and volunteers working in hospitals, in clinics by type of occupation, activity and specialty considering the role of confounders (using university staff cohort, case studies, studying the details of COVID-19 patients who are working in the health sector in terms of the amount and severity of exposure, etc.)	19
Epidemiology	*What is the most accurate estimate of the basic reproduction number (*R*_0_)? Determining the contagiousness of the disease by calculating *R*_0_ and the attack rate	20
Epidemiology	*Which controlling and restraining interventions are effective in reducing the average number of cases inflicted by one individual in a mixed population (susceptible and non-susceptible) [effective reproduction number (Rt)]?	21
Clinical management	*Assessing the effective factors in the sensitivity of infection diagnosis tests with reverse transcription polymerase chain reaction (RT-PCR), including the quality of sampling from the patients, type of sample, sampling time, test site, diagnostic kit, disease severity, etc.	22
Epidemiology	*Evaluating the risk factors for COVID-19 disease infection (case–control study, use of the cohorts present in the country) and evaluation of predisposing factors (socioeconomic status, consumption of opium, different types of drugs and tobacco use including cigarettes and shisha, alcohol, obesity, nutritional status) occupational conditions, comorbidities and immune system diseases, especially HIV-positive people, travel to high-risk areas, etc.	23
Epidemiology	*Evaluation of mortality trends in non-COVID-19 patients before and after the onset of COVID-19 crisis	24
Epidemiology	Evaluation of the short-term and long-term side effects of COVID-19 disease in patients after hospital discharge, monitoring the patients who were not hospitalized	25
Clinical management	*Monitoring and evaluation of all interventions currently underway, including evaluation of the implementation of protocols for the preparation and provision of healthy nutrition for hospital staff (provision of basic materials, kitchen, distribution, quality, staff satisfaction with food, leftover food collection, etc.)	26
Clinical management	*National and provincial studies on diagnostic methods, disease progression, interventions and drugs used, intensive care, consequences of patients with COVID-19 disease to identify differences and the impact of these differences on consequences	27
Clinical management	*The performance of diagnostic and serologic, PCR, laboratory and computed tomography (CT) scan tests in diagnosing COVID-19 disease	28
Infection control	Identify the effectiveness of infection prevention and controls (IPCs) in public settings (use of masks by healthy individuals; precautions for care at home for family members and the community; education; management of corpses)	29
Social science, public health and participation in responding to public health and clinical research	*Assessing the social and psychological damage of COVID-19 at the individual level: examining the impact of lifestyle changes during quarantine and its psychological effects, people’s self-care strategies (psychologically and physically) to maintain their passion for life	30
Clinical management	*Identification and description of specific cases and syndromes of COVID-19 disease and reporting the syndromes, disease progression, performed treatments and effects of drugs and performed treatments	31
Social science, public health and participation in responding to public health and clinical research	Assess the level of anxiety and mental security in children	32
Epidemiology	*The impact of the COVID-19 epidemic on incidence, prevalence and spread of other diseases	33
Social science, public health and participation in responding to public health and clinical research	Identify the best ways to engage the community quickly and regularly and encourage them to participate in clinical trials	34
Social science, public health and participation in responding to public health and clinical research	*Reasons for the spread of alcohol poisoning and its association with the COVID-19 epidemic (are alcoholic drinks which have always been ingestible a problem, or do people mistakenly drink antiseptic alcohol?)	35
Epidemiology	Demographic data analysis of COVID-19 disease, and assessment of Lyme condition, disease severity, disease progression, differences between provinces and cities and related causes [according to demographic data (the use of hospital data can be misleading)]	36
Infectious control	Understanding IPC capacity and perceptions by using behaviour change sciences and social sciences (What are the best ways to convey IPC-related advice? The role of media coverage, home care precautions; maximum IPC decline; barriers and facilitators affecting health personnel capacity; human and ergonomic factors; separation and personal protective equipment (PPE), fatigue due to isolation and PPE?)	37
Clinical management	*Evaluation of the disease progression and disease consequences in specific groups (pregnant mothers, children, the elderly, etc.)	38
Social science, public health and participation in responding to public health and clinical research	*Multidisciplinary and interdisciplinary interventions, randomized control trials (RCTs) in developing behaviours and learning prevention strategies	39
Clinical management	Studies related to the range of manifestations and the clinical profiles of patients in mild and severe cases of the disease	40
Clinical management	*Evaluation of nosocomial infections and disease transmission in hospitals and methods to reduce this type of infection in hospitals	41
Epidemiology	What are the environmental conditions associated with the increased likelihood of transmission (e.g. temperature, humidity, seasonal conditions)? *Accurate study of the impact of weather on disease transmission mainly at the meso and micro level, research on the impact of temperature on the eradication of the disease	42
Infection control	What are the risk factors for virus transmission and disease outbreak among healthcare workers?	43
Social science, public health and participation in responding to public health and clinical research	*Socioeconomic factors affecting the control, infection and treatment of COVID-19 and the impact of sociocultural factors on the global COVID-19 challenge	44
Clinical management	*Statistical modelling and determining the correct method of analysing the information recorded in hospitals in terms of disease consequences	45
Clinical management	*The study of the clinical characteristics, severity and the progression of the disease in specific patients (cardiovascular patients, organ transplants, diabetes, cancers, chronic respiratory diseases, AIDS, immunosuppression, chronic kidney diseases, etc.) at the national and provincial levels	46
Social science, public health and participation in responding to public health and clinical research	*Build an app for people that takes into account the psychological dimensions for suspicious people and others (currently, everyone wants to have a CT scan and COVID test, while it is not really needed)	47
Epidemiology	*Modelling to determine the time of the end of crisis (When will the wards return to their original state? What is the prediction for the unforeseen sudden crisis?) Predicting the course of the epidemic and its trend/using intelligent methods to predict the epidemiological indicators of the disease	48
Epidemiology	*Futuristic models for identifying the needed interventions and preparedness for different epidemic conditions	49
Epidemiology	What is the cause of epidemiological time delays (for example, from the onset of symptoms to the diagnosis of the disease, the onset of symptoms to hospitalization), and what effect do these delays have on epidemic doubling time?	50
Ethics	*Case study of the moral manifestations of the health community in the COVID-19 epidemic	51
Infection control	How important are separation, quarantine and ideal processes in medical care? Co-separation in front of single rooms; costs and resources for co-separation, criteria, principles and cost-effectiveness of quarantine; unintended consequences of quarantine and isolation. Medical care processes and access to responsive health services to minimize contacts	52
Vaccine R&D	*The impact of genetic factors and various interleukins on COVID-19 disease in order to make drugs and vaccines specific to the Iranian population by using the country’s existing bio-banks	53
Infection control	Identifying the importance of PPE and IPC measures and the relative importance of special PPE/IPC measures, type of the mask and eye protection; is it necessary to take precautions against airborne droplets in certain environments (regular care against aerosol production methods), PPE for triage, spatial separation with optimal distance? Comparison of the quality and results of personnel prevention tools (masks, disinfection, clothing, guns, separation, etc.) and their results in different hospitals, reviewing different methods of protecting personnel and the results of such measures in hospitals	54
Epidemiology	Evaluation of susceptibility, infection and the severity of COVID-19 infection in non-COVID-19 patients (all cases of the hospitalized or non-hospitalized populations)	55
Virology, transmission, diagnosis	How can technical deficiencies such as simple immunofluorescence antibody assay (IFA), differential IFA, enzyme-linked immunosorbent assay (ELISA), neutralization assay, substitute neutralization methods including false types and competitive ELISA be addressed?	56
Social science	Assessing people’s views on traditional medicine methods introduced during the COVID-19 disease crisis	57
Epidemiology	What is the relative importance of the virus transmission in asymptomatic or pre-symptomatic individuals? Is there a transmission, and what is its impact? Can asymptomatic vectors transmit the virus? *Assessing the frequency of asymptomatic individuals, retrospective screening of patients for accurate and individualized recording of the mode of disease transmission, assessing the rate of virus transmission in families (father–child, wife–husband, mother–child, brother–sister, identical twins in comparison with fraternal, etc.)	58
Epidemiology	*Using mathematical modelling and determining high-risk groups by using neural network methods and other mathematical models	59
Virology, transmission, diagnosis	What are the tests that determine the point of care (care testing)?	60
Virology, transmission, diagnosis	Identifying methods for diagnosing diagnostic drift (PCR compatibility may change over time due to mutations in probe or primer-binding sites)	61
Virology, transmission, diagnosis	Identification of escape mutants (in the laboratory) and genotypic–phenotypic approaches (to monitor treatment). It is suggested that sequences be prepared from samples of patients from different ages in different phases of the disease [mild, moderate, severe, acute respiratory distress syndrome (ARDS) and deceased patients] and try to investigate the possible relationship between the occurrence of mutations and these phases and finally draw conclusions. Criteria for sample selection and evaluation are listed in separate instructions	62
Epidemiology	Does the infection produce neutralizing antibodies? Are there any antibody-dependent (vaccine) strategies to fight the disease and infection?	63
Virology, transmission, diagnosis	What are the reliable methods for measuring antibodies?	64
Epidemiology	What is the impact of PPE and measures such as maintaining social distancing and wearing a face mask?	65
Virology, transmission, diagnosis	What is the relationship between genetic indicators and reduced phenotypic sensitivity to specific antivirals (more information on virus and host characteristics is needed to predict virus traits or disease severity)	66
Virology, transmission, diagnosis	Are immune indicators related to the prognosis of the disease?	67
Virology, transmission, diagnosis	What are the digital solutions to help the field lab?	68
Virology, transmission, diagnosis	Can cellular immunogenicity be measured with cell-surface substitutes [enzyme-linked immune absorbent spot (ELISpot), etc.]?	69
Epidemiology	What are the causes and conditions that increase the power of transmission? What is their contribution to the spread of the disease?	70
Clinical management	*The studies whose response to preparing nutrition guidelines (what foods boost the immune system) for specific conditions, such as immunodeficient patients and ICU patients, and guidelines for caring for pregnant mothers, including the needed training, diagnosis, determining the necessary visits and determining the necessary ultrasounds to reduce the number of visits, psychiatric counselling and follow-up	71
Infectious control	Identifying transmission methods and transmission duration (which affect the selection of the most appropriate IPC measures and their optimal duration)	72
Virology, transmission, diagnosis	How can the loss of assessing efficiency due to mutations be prevented? This is true of commercially manufactured kits that may not be compatible with intra-organizational PCR speeds and may emit fewer starter/prober sequences. This threat is minimized by developing PCR methods that target protected areas that are relatively stable	73
Epidemiology	Are children less susceptible to COVID-19? If so, why? If they are susceptible but asymptomatic, are they vectors of the virus? Do they transmit the virus?	74
Virology, transmission, diagnosis	What are the sequencing approaches on the patient's bedside and laboratory?	75
Infectious control	Identifying the environmental stability of the virus and effective methods to minimize the role of the environment in its transmission	76
Infectious control	Survival of the virus on surfaces and factors affecting the stability of the virus (e.g. surface type, humidity, temperature, the amount of protein). The effectiveness of various disinfectants for cleaning surfaces around the patient, including the extensive range used in different situations (cleaning body fluids versus regular surface cleaning) and in environments with different levels of resources used	77
Virology, transmission, diagnosis	What technologies should be developed to identify prognostic indicators?	78
Virology, transmission, diagnosis	Can serological specificity and co-stimulation or serological cross-reactivity add value to serological diagnosis?	79
Virology, transmission, diagnosis	Is viral load or viral load pathway (viral load trajectories) related to disease prognosis (knowing this relationship is necessary to create a profile of disease severity)? Due to the lack of quantitative kits on the market at this time, cycle of threshold (Ct) analysis in real-time PCR tests should be the criterion for evaluation	80
Infectious control	*Evaluation of waste management and infectious waste in COVID-19 hospitals	81
Infectious control	*Preparation of evidence-based guidelines for infection control in hospitals (for example, the difference between the persistence of the virus on metal and plastic and its use in hospitals), preparation of personnel protection protocol	82
Virology, transmission, diagnosis	Are there any possible methods for detecting multiple respiratory pathogens?	83
Epidemiology	*Does innate immunity in this disease make sense? That is, can we assume that a percentage of the population, even if they have effective contact, have no chance of infection, or it is very low? What is the nature of this immunity (genetics or cross-immunity with other microorganisms and even other vaccines, etc.)	84
Virology, transmission, diagnosis	What indicators determine the infectivity of the disease (such as discharge-related criteria, and how much the viral load in the upper respiratory tract versus the lower respiratory tract can be a reliable alternative indicator for this purpose)?	85
Infectious control	*Is spraying of disinfectants at the entrances of hospitals and offices effective in eliminating the COVID-19 virus? Which disinfectant and with what density and when is effective?	86
Vaccine R&D	While there is a good understanding of what should be done in a clinical trial at an early stage, fundamental decisions need to be made about the design of clinical trials at a later stage	87
Virology, transmission, diagnosis	Is there an innate immunization in these viruses?	88
Clinical management	Viral kinetic study and pathophysiology of severe COVID-19 disease	89
Infectious control	Identifying all target tissues for virus entry, identifying all body fluids that contain the virus and can transmit the virus (RNA detection against live virus and determination of viral load), importance of airborne transmission and “opportunistic airborne” spread and vertical transmission. The duration of spread and the probability of asymptomatic spread; the ability of the virus to transmit to others through asymptomatic transmission and, if proven, the relative frequency of such transmission events	90
Infectious control	Provide services securely. Electronic monitoring of syndromic signatures of people under supervision and care at home and patients in isolation (e.g. the use of point-of-care sensors and wearable monitoring and artificial intelligence support)	91

In the epidemiology domain, the issue of disease outcome was added to the knowledge needs, although it overlapped significantly with disease susceptibility and severity. In the sensitivity section, a range of risk factors were specifically mentioned, in which illicit drug abuse and shisha (water pipe) smoking received special attention. In this domain, another knowledge gap was added as a sub-domain, which was titled the epidemiological dissemination pattern of the COVID-19 virus and its consequences in the country.

The domain of clinical management is more affected by the context and the access to facilities and equipment, and its knowledge needs were identified as context-specific, such as the implementation of clinical practice guidelines, the quality and standards of diagnostic tests, the changes in the course of the disease in various service centres and the needed information to compile general and comprehensive clinical guidelines for high-risk groups.

In the infectious control domain, knowledge needs related to waste management in hospitals as well as the method of infection control at the level of the city, organizations and communities were identified.

Another point that was identified in this regard as to cultural conditions was the attention that was paid to following the hygienic guidelines for issues related to the deceased while considering the cultural issues.

In the domain of candidate treatment and vaccine R&D knowledge, an important knowledge need was suggested: “genetic studies of the population of patient profiles and using the collecting biobanks, which are applicable in targeting prevention and treatment”. It was also suggested that an observational evaluation of the treatment methods used in the country and its changes and consequences be examined using data science and machine learning approach.

In the domain of ethics, in the prioritization presented by GloPID-R, the main focus was on ethical considerations in COVID-19 research, while the subject proposed in this study was the examination of the manifestations of ethics in society, which is, of course, very context-specific.

In the social sciences domain, research questions were identified in the two sub-domains of “care, access and health system” and “public health and participation in response to public health and clinical research”. All these recognized questions were new.

The results of prioritizing the identified research questions are presented in Table 2. As shown in the table, the first 12 priorities are the knowledge needs identified in this study, and all belong to the two fields of epidemiology (transmission dynamics) and social sciences.

All identified challenges in the field of social sciences were about HPSR. As shown in Table 3, multiple challenges were identified in all subareas of this framework. It is worth emphasizing that this study was completed during the first 4 months of the COVID-19 epidemic in Iran, and all these challenges might be time-dependent. Since that time, some challenges have been addressed, but research is needed to find the best intervention to reduce these challenges for similar future crises.

**Table 3:** Iran’s health system challenges in responding to COVID-19

**Monitor health status to identify health problems**
1. The weakness of the country’s surveillance system in the screening and diagnosis of cases in the country
2. The weakness in on-time detection of the virus in the country and lack of a foresight for it. Weakness in capacity-building for epidemiological crisis management in the country, lack of modelling about health system's exposure to an infectious disease in advance considering the high probability of the occurrence of such a problem based on the symptoms and target groups and complications of the disease, medication and equipment and the duration of COVID-19 treatment
3. Non-institutionalization of International Health Regulations (IHR) in the country
4. Weakness in patient detection and identifying travellers entering the country from infected countries (interventions related to detecting high-risk cases, training them, quarantining and tracking their contacts)
5. Weakness in benchmarking the similar experiences of other countries
**Diagnose and investigate health problems and health hazards**
1. Weakness in capacity-building and preparation of comprehensive health centres to deal with the epidemic
2. Weakness in support capacities and diagnostic, care and treatment facilities
**Inform, educate and empower people about health issues**
1. Weaknesses and limitations in communicating with society and the exchange of information, lack of planning to carry out activities to gain the trust of the people and lack of specific programmes based on community participation
2. Weakness in planning and preparing society for the health and socioeconomic consequences of the disease
3. Weakness in the management of information related to the disease and the lack of a registration system for the disease in both preventive sector and treatment sector and hospitals, as well as governmental and nongovernmental sectors
4. Weakness and slowness in notification, education and media for people (different groups) and different classes
5. Unreal cyberspace with false news, therapeutic rumours, self-treatment and herbal drugs
6. Public concerns because of various reasons, including the concern of different trades over getting sick during work, concern over the lack of knowledge about the disease and concern over the inadequate notification about at-risk people
7. People not paying attention to protection against the disease and considering instructions, and teleworking
8. Emergence of a range of feelings of hopelessness, learned despair and changing attitudes towards the meaning of life in society
9. Increase in methanol poisoning for preventing COVID-19
10. Many people attending medical centres and people flocking to buy masks, sanitizers and gloves and its shortage and the existence of a black market
**Mobilize community partnerships to identify and solve health problems**
1. Lack of appropriate and on-time advocacy in the country’s policy-makers for preparedness of the country at the beginning of the epidemic and weakness in multisectoral cooperation
2. Lack of comprehensive planning to use the capacity and potential of the private sector and social institutions
**Develop policies and plans that support individual and state-wide health efforts**
1. Weakness in crisis management and designing a comprehensive plan to combat and control the epidemic and formulate it so that the interventions are known separately for each stage of the epidemic and the necessary predictions for different scenarios
2. Delay in involvement of the health and prevention sector in the management of COVID-19 and the lack of attention to the contact tracking system and more attention to the treatment sector at the beginning of the epidemic
3. Delay in all necessary actions such as quarantine and social distancing
4. Insufficient use of scientific evidence (economic calculations, disease burden, etc.) in policy-making to develop intervention to cut the chain of transmission
5. Lack of a unified and specific approach to hospital management in terms of crisis management (the problem was financing in service-providing facilities due to lack of elective operations and income-generating services) and the disorientation of other patients both in hospitals and in offices and clinics
6. Lack of updating diagnostic guidelines (sensitivity and specificity of tests) and treatment based on new information and disagreement
7. Weakness in digital employment and the lack of a telemedicine system
8. Lack of attention and planning for spending the patient’s recovery period after discharge
9. Lack of appropriate policy-making for hospitals in order to divide the admission of COVID-19- and non-COVID-19infected patients
10. Relative shortage of hospital equipment, protective equipment, medicine, diagnostic kits and the existence of a medicine and laboratory black market
11. The impossibility of allocating subsidized masks and sanitizers to the community and various social and industrial groups
12. Public transportation problems due to restrictions and congestion in them
13. Problems of waste disposal, especially hospital waste, the problem of keeping corpses in hospitals
14. Weakness in the implementation of protocols such as disinfection of schools, public centres, prisons, rehabilitation centres, public health services, bakeries, banks, welfare centres, etc.
**Enforce laws**
1. Not using the capacity of legislative bodies for the approval of effective measures
2. Weakness in the accountability structures of the Iranian health system (structural, legal, executive) that was more apparent during the crisis
**Link people to needed personal health services and assure the provision of healthcare when otherwise unavailable**
1. Weak laboratory facilities, delays in receiving test kits and preparation of laboratories
2. Lack of attention to determining the norms and per capita of protective equipment and sanitizers required per person in each of the preventive and treatment sectors due to the existing shortcomings and also the economic situation
3. People’s unwillingness to donate blood during an epidemic
4. Failure in routine vaccination of children due to fear of attending health centres
**Assure a competent public and personal healthcare workforce**
1. Shortage of treatment staff
2. Burnout, job stress, physical and mental fatigue and lack of motivation of the health workforce which dealt with COVID-19
3. Relative shortage of personal protective equipment for health workforce and lack of a single protocol on personnel protection in all hospitals
4. Lack of knowledge and empowering of health staff in identifying and dealing with COVID-19, especially at the beginning of the epidemic at the different levels of the health system
5. Lack of an integrated view toward the health workforce as a team facing the risk and the dissatisfaction among lower-level workers (such as supportive services personnel), especially in the treatment sector
6. The high level of infection and death among medical staff
**Evaluate effectiveness, accessibility and quality of personal and population-based health services**
1. Weakness in the implementation of waste disposal protocols
2. Weakness in providing psychological support to patients infected by COVID-19 and their families, the incomplete mourning of the bereaved families and lack of physical sympathy with mourners and consoling them
**Contextual and general challenges**
1. The lack of clarity in professional boundaries and at the same time lack of teamwork in Iran, which was more apparent better during the crisis
2. Weakness in the proper management of ongoing studies for COVID-19
3. The unavailability of COVID-19related data for researchers
4. The direct and indirect high economic burden of the disease in the face of international sanctions
5. Socioeconomic harms include:
a. Economic consequences: reduction of the country's foreign exchange earnings, economic consequences for various guilds and on exports and imports, as well as cancellation of flights from other countries to Iran
b. Reduction of social capital and the negative effects of stress on families and children
6. An increase in water consumption in the country and the reduction of water quality in some cities

## Discussion

The present study was designed to answer two main questions. First, to identify the knowledge needs to respond to the current COVID-19 epidemics and future crises. We also aimed to investigate how to adapt international organizations’ research priorities in the COVID-19 setting in each country. These results were important in this situation because countries like Iran have limited resources for research, and it is important that these limited resources are allocated to fill the knowledge gaps and clarify the country research map during the COVID-19 epidemic.

According to the results of this study, the knowledge needs, in addition to what was identified in the GloPID-R’s road map, are of two categories. The first group was knowledge needs that are not context-specific but its responses are context-specific (such as evaluating the implementation of standard international protocols). In addition to the two areas of “virology, transmission, diagnosis” and “animal and environmental research”, knowledge needs were identified in other areas in which research is required in every country in order to produce the knowledge it needs.

This issue is fundamental in the social sciences and the sub-field of HPSR. Therefore, it is recommended that a suitable framework be added to the list of knowledge needs in international research priority-setting, but countries must certainly conduct specific research in order to find the answers.

We also found that one of the main priorities in any country is implementing protocols and interventions at the individual, organizational and societal levels. In fact, these types of action research and participatory research projects should be considered as priority by funding organizations.

The second group is the context-specific knowledge needs, such as examining the causes of differences in symptoms, disease severity and development of COVID-19 in different parts of the country or by social variables that determine health. Therefore, countries should pay special attention to identifying their specific knowledge needs according to the regions (provinces).

There are two studies which investigated the applicability of WHO/GloPID-R after a few months at the beginning of the COVID-19 pandemic, but both were found to place greater emphasis on social science and the temporal nature of research priorities [10, 11]. Our study confirmed their conclusion by adding the need to have a special theme for HPSR.

## Strengths and limitations

This research priority-setting was ordered by a funding agency, and this was the most important strength. In the systematic review of research priority-setting in Iran, it was found that a lack of implementation plans for research priority-settings was a problem, but the results of this study were used by NIMAD to fund research [12]. One of the strengths of this study was the use of existing challenges in responding to the COVID-19 epidemic to identify context-specific knowledge needs, which was done for the first time in this study. Although the involvement of patients and the community in research priority-setting is routine, identifying the challenges in responding to a crisis in order to find knowledge needs is the dominant feature of this study. Dr Glison and her colleagues have clearly described why we need HPSR to respond to the existence of the COVID-19 epidemic and transform it into a resilient health system for future crises. They have explained the essential features of including a systematic approach that is multidisciplinary and context-specific. One approach to lead HPSR and meet this need is to start from “challenges” [13], challenges of countries in having the ideal response to the COVID-19 epidemic. The advantages of this approach are as follows:

1. When we use “challenges” to define HPSR priorities, we have chosen a context-specific approach. Some challenges are specific to the context and need context-specific solutions. For some challenges which are common among countries, their solution would be unique for each context.
2. To address the challenges in response to the COVID-19 epidemic, it is inevitable to produce an actionable message to solve challenges. In other words, we should clarify for policy-makers what should be done, by whom, where and when. It is necessary to conduct interdisciplinary and multidisciplinary research to produce an actionable message. Research with a narrow question cannot address the challenges alone.
3. When we consider “challenges’’ as the starting point for identifying HPSR questions, we have chosen the system thinking approach. We have to think about the governance, financial and delivery structure of the health system, implementing current policy and programmes, and contextualizing solutions according to the health system and political system considerations in each country.

Four research questions have to be answered to solve each challenge: (1) What is the magnitude of the challenge? (2) What factors have created the challenge? (3) What is the mechanism of the impact of the challenge in responding to the epidemic? (4) How should the identified challenge be solved? Therefore, countries should design an active and up-to-date system to identify their challenges in determining the required research priorities and direct research funding in that direction.

Also, the use of the methodology of accountability for reasonability (A4R), which is used to ensure the legitimacy of decisions in health based on the five pillars of “relevance”, “publicity”, “revision and appeals”, “empowerment” and “enforcement”, was a strength of this study and could be suggested for future actions in this field [14].

One limitation in this study was the unavailability of policy-makers to do in-depth interviews. This limitation was expected; alternatively, it is suggested to participate in the policy-making meetings as a broker [4], but it was not possible in this study.

## Conclusions

The COVID-19 pandemic has led to an increase in the demand for healthcare services in all countries. Due to a lack of knowledge and the urgency of the situation, several research questions were raised to increase the effectiveness and efficiency of public health programmes to control the disease. Therefore, identifying knowledge needs and prioritizing research questions was crucial to responding to this demand appropriately. This prioritization can be done globally, but some of the research priorities are context-specific and may vary by regional needs. We found that the epidemiology (transmission dynamics) and social sciences domains were considered as the main priorities during the first few months of the COVID-19 epidemic in Iran. It can be concluded that in the global health crises, in order to manage research resources better and to make evidence-informed decisions, global knowledge gaps must be localized in domains, which are highly affected by political, economic, social and cultural characteristics of each country.

## Supplementary Information

**Additional file 1:** The methodological details of Multiple Criteria Decision Making Model (MCDM).

## Data Availability

Detailed data for each step are available and can be provided upon request.

## Abbreviations

GloPID-R: Global Research Collaboration for Infectious Disease Preparedness
NIMAD: National Iranian Medical Research Development
HPSR: Health policy and systems research
