# Supplementary material for: Knowledge gaps and national research priorities for COVID-19 in Iran
Source: Health Res Policy Syst. 2022 Mar 2;20:25. doi: 10.1186/s12961-021-00805-y (PMC8889392; doi:10.1186/s12961-021-00805-y)
Supplement: Supplementary file 1 — Additional file 1: The methodological details of Multiple Criteria Decision Making Model (MCDM). [file 12961_2021_805_MOESM1_ESM.docx]

**Additional file 1. The methodological details of Multiple Criteria Decision Making Model (MCDM)**

For prioritizing research questions Multiple Criteria Decision Making Model (MCDM) which is quantitative method was used. In this model after forming the decision matrix, knowledge of the relative importance of decision criteria is necessary to simplify the decision process and prioritize research questions. The relative importance of the criteria is obtained by determining their relative weights. There are various methods for determining the weight of the criteria; Shannon entropy was used in this study due to its greater ease and the fact that it does not require complex pair wise comparisons. The entropy decision matrix can also show the scattering of the values of the criteria. The higher the scattering of the values of a criteria in the research questions, the more important that criteria is in the decision. Because in decision making more attention is paid to the dissimilarity of research questions. Finally, the weights were obtained. Then, for final prioritization, the simple weighting method was used, which is the simplest method in multi-criteria decision making. In this method, which is also known as the weighted linear composition method, after de-scaling the decision matrix, by using the weight coefficients of the criteria, the weighted de-scaled decision matrix is obtained and according to this matrix the score of each research question is calculated. If there are *n* criteria and *m* research questions in a multi-criteria decision problem, the steps of the method in order to select the best research question by using the simple weighting method are as follows:

Decision matrix formation: According to the number of criteria, number of research questions and evaluation of all research questions for different criteria, the decision matrix is formed as follows:

$$X=\left[ \begin{matrix} x_{11} & \cdots& x_{1n} \\ \vdots& \cdots& \cdots\\ x_{m1} & \cdots& x_{mn} \end{matrix} \right]$$

In which x_ij is the function of the research question (i = 1,2,…, m) in relation to the criterion j (j = 1,2,…, n).

De-scaling the decision matrix: In this step, we try to turn the criteria with different dimensions into dimensionless criteria and define the R matrix as follows:

$$R=\left[ \begin{matrix} r_{11} & \cdots& r_{1n} \\ \vdots& \cdots& \cdots\\ r_{m1} & \cdots& r_{mn} \end{matrix} \right]$$

The following equations are respectively used to de-scale the positive and negative criteria:

$$r_{ij}=\frac{x_{ij}}{\begin{matrix} max \\ i \end{matrix}\left\{ x_{ij} \right\}}$$

$$r_{ij}=\frac{\frac{1}{x_{ij}}}{\begin{matrix} \begin{matrix} max \\ i \end{matrix} & \left\{ \frac{1}{x_{ij}} \right\} \end{matrix}}=\frac{\begin{matrix} min \\ i \end{matrix}\left\{ x_{ij} \right\}}{x_{ij}}$$

It is observed that negative criteria are reversed. Another group of researchers multiplied the negative criteria by -1 in this method and converted it into positive methods.

Determining the weight vector of criteria: At this stage, according to the coefficient of importance of different criteria in decision making, the weight vector of criteria is defined as [w_1, w_2, ⋯, w_n].

Selecting the best research question: At this stage, the best research question is obtained from the following relation.

$$A^{*}=\left\{ A_{i} \right.\left| \begin{matrix} max \\ i \end{matrix} \right.\left. \sum_{j=1}^{m} w_{j}r_{ij} \right\}$$

Finally, based on the evaluation of experts and also the weights obtained from Shannon entropy method, the following research priorities were extracted.
